# Effect of Chemical Treatments on the Mechanical Properties of Jute/Polyester Composites

**DOI:** 10.3390/ma17102320

**Published:** 2024-05-14

**Authors:** André Luis Lima Flores, Agnė Kairytė, Jurga Šeputytė-Jucikė, Sylwia Makowska, Alessandra Lavoratti, Rafael de Avila Delucis, Sandro Campos Amico

**Affiliations:** 1 Postgraduate Program in Mining, Metallurgical and Materials Engineering, Federal University of Rio Grande do Sul, Porto Alegre 91501-970, Brazil; andrelflores@gmail.com (A.L.L.F.); alelvt@gmail.com (A.L.); amico@ufrgs.br (S.C.A.); 2 Laboratory of Thermal Insulating Materials and Acoustics, Institute of Building Materials, Faculty of Civil Engineering, Vilnius Gediminas Technical University, Linkmenų St. 28, 08217 Vilnius, Lithuania; jurga.seputyte-jucike@vilniustech.lt; 3 Institute of Polymer and Dye Technology, Faculty of Chemistry, Lodz University of Technology, Stefanowskiego 12/16, 90-924 Lodz, Poland; sylwia.czlonka@edu.p.lodz.pl; 4 Postgraduate Program in Materials Science and Engineering (PPGCEM), Technology Development Center, Federal University of Pelotas (UFPel), Pelotas 96010-610, Brazil; rafael.delucis@ufpel.edu.br

**Keywords:** jute fiber, polyester resin, alkali treatment, hydrogen peroxide, peracetic acid, RTM

## Abstract

Natural fiber composites have been extensively studied for structural applications, with recent exploration into their potential for various uses. This study investigates the impact of chemical treatments on the properties of Brazilian jute woven fabric/polyester resin composites. Sodium hydroxide, hydrogen peroxide, and peracetic acid were utilized to treat the jute fabrics, followed by resin transfer molding (RTM) to form the composites. Evaluation included water absorption, flexural strength, tensile strength, and short-beam strength. The alkaline treatment induced changes in the chemical composition of the fibers’ surface. Chemical treatments resulted in increased flexural and short-beam strength of the composites, with no significant alterations in tensile properties. The hydrogen peroxide treatment exhibited lower water absorption, suggesting its potential as a viable option for enhancing the performance of these composites.

## 1. Introduction

Natural fibers have been utilized in composite materials since ancient times, especially for construction purposes [1]. With the growing awareness of the need to limit non-renewable resource use, researchers are exploring environmentally friendly composite materials using plant matrices to replace synthetic fibers. This shift aims to reduce dependence on oil-derived products and mitigate negative environmental impacts. Composite materials reinforced with natural fibers are increasingly used in engineering applications, especially in automotive, civil, furniture, and sporting goods, albeit mainly in non-primary structural components [2,3].

Natural fibers, also known as lignocellulosic fibers, consist mainly of cellulose, hemicellulose, and lignin, along with minor components like waxes, pectins, and extractives [4]. Cellulose forms crystalline microfibrils, while hemicelluloses include various monosaccharides [5]. Lignin, an amorphous polymer, arises from enzyme-initiated dehydrogenative polymerization. Pectin, crucial for fiber binding, varies in plant tissues, especially in fruit peels and gums [6]. Waxes contain water-soluble alcohols, including phenolic, oleaginous, and stearic acids [7].

Jute fiber is one of the most used natural fibers in composites. It belongs to the family of the Tiliaceae, with around 40 Capsularis species of jute. The main constituents of jute fiber are 61–73% of cellulose, 13.6–23% hemicellulose, and 12–16% lignin, along with small amounts of pectin, fats and waxes usually present on the fibers’ surface. Jute fibers also have a density of 1.5 g.cm-3 and a Young’s modulus of 15–30 GPa [8,9]. They have demonstrated a potential for enhancing several important properties and sustainability in various applications. Generally, jute fibers find applications in rudimentary and economical textile goods. Enhancing the characteristics of jute to align with high-value and technical textiles could yield significant benefits not only in terms of cost but also for the environment [10].

In Brazil, jute plays a significant role, adapted genetically to thrive in the humid soils of Northern Brazil, particularly along the Amazon and Solimões rivers. Covering approximately 15 cities, these plantations contribute to the employment of around 20 thousand workers. Notably, the Brazilian jute production chain produces essential end-products such as yarns, fabrics, and bags, particularly for coffee packaging. As highlighted in Flores et al. [11], exploring new applications that increase the value of jute fibers could bring substantial socio-economic benefits to marginalized communities involved in jute cultivation across different countries.

In order to enhance the adhesion at the interface of natural fiber composites, a number of approaches can be taken. Chemical treatments on natural fibers have been an effective way to clean and enhance the fibers’ surface, increasing the adhesion at the interface by means of altering the surface roughness or even partly removing some waxes on these fibers [12]. Several chemical treatments have been reported in the literature, the most used ones being mercerization, an alkali treatment usually performed with sodium hydroxide (NaOH), hydrogen peroxide, and some acidic treatments [13]. This also includes coupling agents, such as maleic anhydride and silanes [14].

In the study conducted by Wang et al. [15], an investigation centered on the impact of chemical treatments on the composition, structure, and properties of jute fibers, alkali scouring and hydrogen peroxide bleaching were found to effectively eliminate non-cellulose materials, influencing changes in fineness and moisture regain. The treated samples exhibited increased crystallinity indices compared to raw jute, consequently affecting mechanical properties. Bleaching, in particular, significantly influenced brightness and whiteness indices, leading to a substantial reduction in the yellowness index of bleached jute fibers. Recent studies have focused on composites reinforced with chemically treated jute, indicating significant scientific interest in this area. Moreover, review articles have recently been published, shedding light on some key effects observed in these composites [14]. This underscores the growing scientific attention and exploration of chemically treated jute-reinforced composites.

The mechanical properties of jute/polyester composites have been extensively explored through various chemical treatments, aiming to enhance their performance for diverse applications. Several studies have investigated the effects of different chemical treatments on the properties of jute fibers and their composites. Wang et al. [10] focused on acid and alkali pretreatments, affecting void formation and mechanical properties in jute fiber/epoxy composites, while Shahinur et al. [16] explored rot-retardant, fire-retardant, and water-retardant treatments, observing altered thermal behavior. Bulut and Aksit [17] highlighted the importance of oxidative treatments for enhancing interfacial adhesion in jute/polypropylene composites. Sajin et al. [18] emphasized the role of alkali-treated jute fiber length on composite properties. Siddika et al. [19] investigated the mechanical properties of jute/coir fiber-reinforced hybrid polypropylene composites, showing improved properties with increased jute content and alkali treatment. Despite the abundant literature on composites with natural fibers, few studies focus on jute fabric treatments for thermosetting composites.

In summary, natural fibers like jute offer sustainable alternatives for composite materials, addressing the need to reduce reliance on non-renewable resources. While challenges such as fiber heterogeneity and moisture absorption persist, chemical treatments show promise in enhancing fiber compatibility and performance. By leveraging various treatment methods such as alkali scouring, bleaching, and oxidative treatments, researchers have demonstrated significant enhancements in fiber quality and compatibility with composite matrices. This study uniquely contributes by conducting a comparative analysis of different chemical treatments applied to jute fibers, aiming to improve both the mechanical and hygroscopic properties of fabric-reinforced laminated composites. The investigation employs resin transfer molding (RTM) to produce jute fiber fabric composites, evaluating changes in adhesion, mechanical performance, and water absorption induced by the various treatments.

## 2. Materials and Methods

### 2.1. Materials

Unidirectional jute fabrics were obtained from Castanhal Textile Company with a yarn diameter of approximately 0.3 mm. According to the manufacturer, the jute yarns are comprised of 85% cellulose content with a tensile strength of 90 kPa and a weight of 239 g/m². The levels of acid-insoluble lignin, acid-soluble lignin, ethanol/toluene-soluble extractives, ash, and holocellulose (remaining to total 100%) were 9.10% ± 0.68%, 1.91% ± 0.42%, 2.75% ± 0.13%, 0.296% ± 0.05%, and 86.01% ± 1.05%, respectively. To obtain these values, the methods described in Delucis et al. [20] were also employed in the present study. The unsaturated isophthalic polyester resin, commercially known as Arazyn 50502 T10, was chosen due to its good surface finish, mechanical properties, and low cost. It was acquired commercially from Fiberglass located in Porto Alegre, Brazil. The initiator used was methyl ethyl ketone peroxide (MEKP), commercial name Butanox 50, in a proportion of 0.1 parts per hundred resin (phr). The reagents sodium hydroxide, hydrogen peroxide, and peracetic acid were used as received.

### 2.2. Fiber Treatment

The jute fabrics, totaling 2 kg, were subjected to three different chemical treatments: 2% sodium hydroxide (NaOH), 30% hydrogen peroxide (H_2_O_2_), and 2% peracetic acid (C_2_H_4_O_3_). The choice of NaOH, H₂O₂, and C₂H₄O₃ was based on their known effectiveness in modifying the chemical composition of natural fibers and enhancing their compatibility with polymer matrices. Additionally, the concentrations of NaOH, H₂O₂, and C₂H₄O₃ used in the treatments were determined based on previous studies [12,21,22]. The fabrics were immersed in the respective solutions for 10 min at room temperature (20–25 °C) and then rinsed with distilled water until reaching a neutral pH. Subsequently, the fabrics were dried in a circulation oven at 50 °C for 2 h.

### 2.3. Composites Molding

To determine the number of layers to be used, compression was applied to 8, 7, 6, and 5 layers of dry fabric at 0.1 MPa, with 5 being the maximum number of layers, ensuring that the thickness of the fabric assembly did not exceed 2.5 mm. This thickness corresponds to the cavity thickness of the mold used for resin transfer molding (RTM) of the composites.

For the RTM process, a steel mold with dimensions of (300 × 300) mm was employed. The top of the mold was made of 10 mm tempered glass. The mold was internally coated with a semi-permanent release agent (Chem-Trend brand) and connected to an air compressor, which, on the other end, was linked to a pressure vessel. Fabrics were cut, dried (at 50 °C for 2 h), weighed, and positioned inside the mold according to the desired stacking sequence for each laminate. The closing pressure was adjusted by the torque level applied to the mold sealing screws, reaching approximately 10 N·m. This mold closing pressure resulted in a fiber volume fraction (Vf) of approximately 24–27%. This corresponds to a ratio of mass of resin to mass of fabric in the composites ranging from approximately 0.76 to 0.73.

The resin, manually mixed with the catalyst at room temperature for 3 min, was then placed inside the pressure vessel and forced to internally permeate the mold due to a pressure difference of 140 kPa applied by the air compressor. The resin inlet was positioned in the center of the mold, with the outlet occurring from four corners. The mold filling in each molding cycle took approximately 40 min. The composites were cured at room temperature for 24 h and post-cured at 60 °C for 4 h using the aforementioned oven.

### 2.4. Characterization of the Jute Fiber and of the Composites

The changes on the fibers’ surface were evaluated by Fourier-transform infrared spectroscopy in Perkin Elmer SPECTRUM 1000 equipment in a range of 4000 to 500 cm^−1^. The spectra were obtained by total attenuated reflectance (ATR). The density (ρT) of the composites was determined according to ASTM D792. Water absorption was determined according to ASTM D570-10. Scanning electron microscopy was performed in Zeiss EVO MA10 equipment with an acceleration voltage of 10 kV.

Tensile strength testing was performed according to ASTM D3039 with a total of 7 test specimens per sample, with dimensions of 170 mm × 25 mm × 3 mm. The testing speed was 2 mm·min^−1^ until rupture. The longitudinal and transversal deformation/elongation were obtained using a video extensometer.

Three-point flexural testing was performed according to ASTM D7264 using 7 test specimens with dimensions 127 mm × 12.7 mm × 3 mm at a constant crosshead speed of 1 mm·min^−1^ until a deflection of 5% in relation to the length of the sample was reached, keeping a constant span/thickness ratio of 16:1.

Short-beam strength testing was performed according to ASTM D2344, with 6 test specimens of 18 mm × 6 mm × 3 mm at a crosshead speed of 1 mm·min^−1^ until rupture. An Instron 3382 testing machine with a load cell of 5 kN was used for tensile, flexural, and short-beam strength testing.

The numerical data obtained in this study were separated in groups based on the studied factors and tested according to the normality and homogeneity of the variances using Shapiro–Wilk and Levene testing, respectively. To compare the studied groups, one-way analysis of variance (one-way ANOVA) was performed, followed by least significant difference (LSD) Fischer testing within a significance level of *p* < 0.01 (1%).

## 3. Results and Discussion

**Figure 1:**
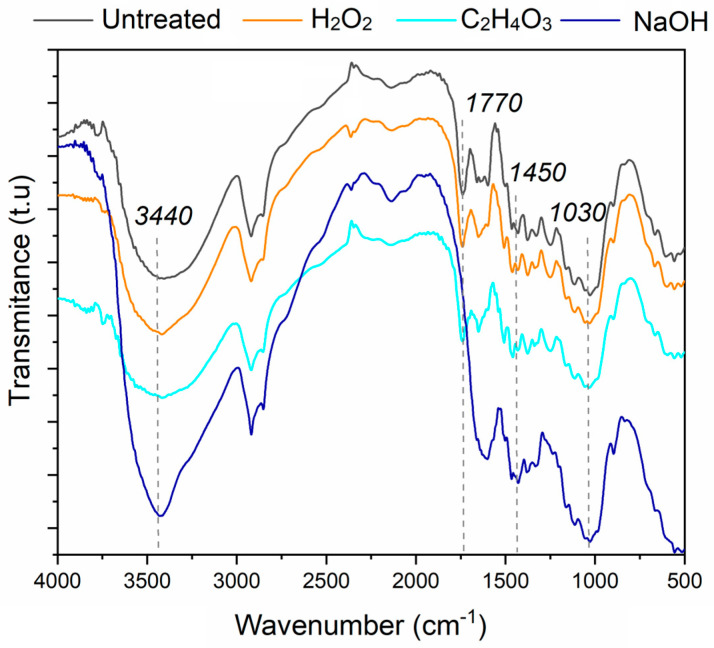
FTIR spectra of the jute fibers with different treatments.

Figure 1 shows the FTIR results for the neat and treated jute woven fabrics. The band at 3440 cm^−1^ is related to the presence of free hydroxyl groups of the fibers’ cellulosic structure [23]. The sharp absorption peak at 3440 cm⁻¹ in the infrared spectrum for the NaOH-treated material indicates changes in hydrogen bonding, possibly due to the removal of hydroxyl groups during the treatment process. These groups can be present in cellulose and hemicellulose. The peak at 1770 cm^−1^ is associated with the C=O stretching vibration and stretching of the ester linkage in hemicelluloses or lignin [24]. At 1450 cm^−1^, the peak is related to C-H vibrations [25]. The peak at 1030 cm^−1^ is associated with the stretching vibration of O-C-O present in cellulose and hemicellulose, and the C-O and C-C stretching vibrations [26].

For the NaOH-treated fiber, the peak at 1770 cm^−1^ was not present, from which it could be inferred that some of the lignin was removed from the surface of the jute fibers. This observation supports the efficiency of the alkali treatment in eliminating loosely bonded structures, as reported by Jo and Chakraborty [24]. Their findings align with the current study, indicating that the alkali treatment effectively removes hemicellulose and lignin from the jute fibers’ surface. This removal is crucial in the context of jute/polyester composites, as it enhances the compatibility between the jute fibers and the polyester matrix, leading to improved adhesion and, consequently, enhanced mechanical properties of the composite material. The improvement in compatibility between jute fibers and polyester resin after NaOH treatment is indeed related to specific functional groups present in the resin. The removal of lignin during the NaOH treatment results in a cleaner fiber surface, free from contaminants, which promotes better adhesion between the jute fibers and the polyester matrix. The functional groups present on the surface of NaOH-treated fibers can interact more efficiently with the functional groups of the polyester resin, such as hydroxyl (-OH) and carbonyl (C=O) groups, thereby enhancing interfacial adhesion and improving the mechanical properties of the composite.

The peak observed at 2250–2500 cm⁻¹ for H₂O₂ and NaOH treatments in Figure 1 may be attributed to the presence of residual peroxides or other functional groups resulting from the treatment process [27]. In summary, the FTIR spectra analysis reveals the intricate changes in the composition and structure of jute fibers after different chemical treatments, underscoring the importance of these treatments in tailoring the properties of jute fibers for optimal performance in jute/polyester composites.

**Figure 2:**
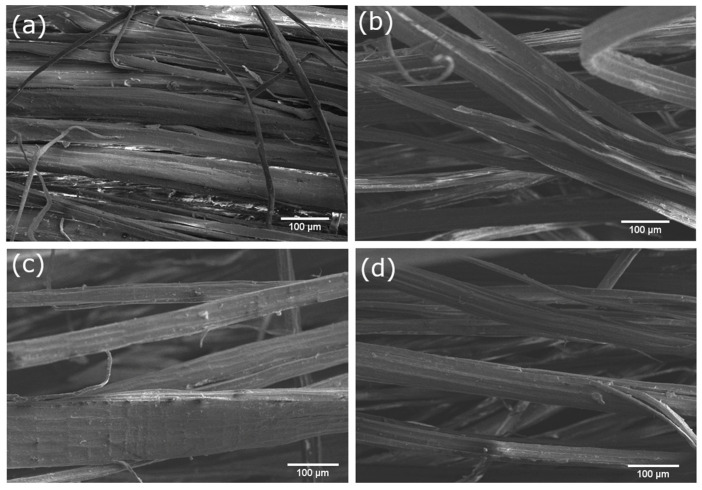
SEM micrographs of jute fibers: (**a**) neat jute; (**b**) NaOH treatment; (**c**) hydrogen peroxide treatment and (**d**) peracetic acid treatment (400×).

SEM micrographs of the jute fibers are presented in Figure 2. The jute fibers in Figure 2a appear to be bundled together and not as separated from each other as the fibers after the chemical treatments employed in this study. This could contribute to the improvement in the aspect ratio of the fibers—i.e., the ratio between the length and the diameter. With an optimum concentration of a chemical treatment, the diameter of the fiber is decreased, resulting in better adhesion due to increased aspect ratio [28].

Following chemical treatments, such as NaOH treatment (Figure 2b), hydrogen peroxide treatment (Figure 2c), and peracetic acid treatment (Figure 2d), a slight increase in surface roughness is evident. This change can be attributed to the removal of waxes, oils, and dirt from the fibers’ surface, contributing to an overall improvement in adhesion between the fibers and the polyester matrix. Notably, the treated fibers appear more separated than the neat jute fibers, suggesting that the chemical treatments have led to a more individualized and dispersed fiber arrangement. This enhanced separation can contribute to an improved aspect ratio, as individual fibers are more effectively incorporated into the composite matrix.

Even though the FTIR results may not indicate significant changes in the chemical composition of the fibers’ surface, the observed morphological modifications could play a crucial role in enhancing the mechanical performance of jute/polyester composites. Specifically, the reduction in fiber diameter, stemming from optimal chemical treatments, may lead to increased aspect ratios, fostering better adhesion between the fibers and the polyester matrix.

**Figure 3:**
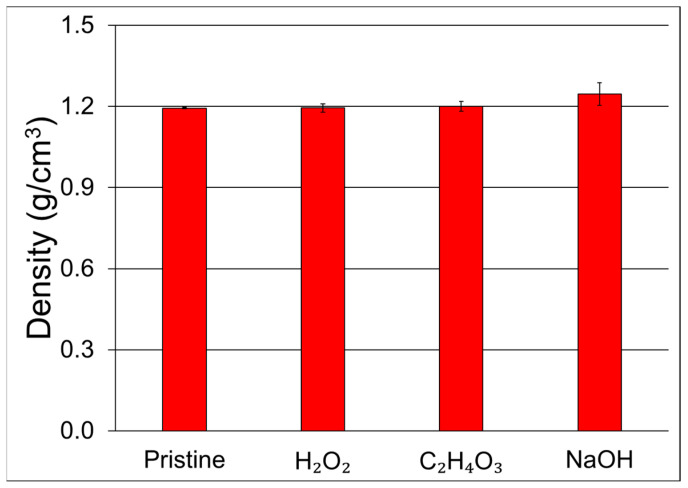
Density of the jute/polyester composites.

The density measurements presented in Figure 3, conducted using Archimedes’ method, provide insights into the overall mass per unit volume of the jute/polyester composites and are crucial for understanding the material characteristics. The results indicate that there is no significant difference in density among the various composite samples, as evidenced by the overlapping values within the standard deviation. All recorded values fall within the narrow range of 1.2 g/cm³.

This consistency suggests that the chemical treatments applied to the jute fibers did not cause a substantial alteration in the overall density of the resulting composites. Comparisons with the existing literature further support these findings. The observed density range of 1.1–1.2 g/cm³ for the jute/polyester composites aligns with similar composite systems reported in previous studies [29]. The consistency in density across different samples and conformity with literature values indicate that the chemical treatments did not significantly impact the mass per unit volume of the composites.

**Figure 4:**
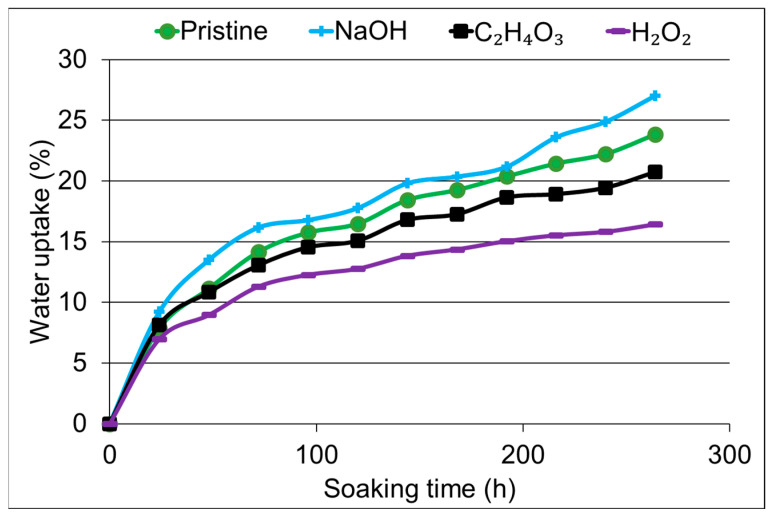
Water absorption results of the composites until saturation.

The water absorption results depicted in Figure 4 provide crucial insights into the interaction between chemical treatments, surface modifications, and the hydrophilic behavior of the jute/polyester composites. Notably, the NaOH-treated composites exhibited the highest water absorption, in contrast to the other treatments, including the neat or pristine fibers. This observation aligns with the significant increase in intensity of the band at 3440 cm^−1^ in the FTIR spectra, which is associated with OH- groups on the fiber surface, arising from either exposed cellulose or free water. The intensified band suggests a higher presence of hydrophilic groups, contributing to increased water absorption. This trend is consistent with previous studies on natural fibers, where increased hydrophilicity, marked by exposed OH- groups, is linked to higher water absorption [30,31].

Conversely, composites treated with hydrogen peroxide and peracetic acid exhibited the lowest water absorption. Despite the absence of notable changes in the FTIR spectra for these treatments, SEM micrographs revealed surface roughness on the fibers. This introduced roughness could enhance adhesion between the fibers and the polyester matrix, potentially creating a barrier that limits water ingress. The reduction in water absorption observed in these composites suggests that the surface modifications induced by hydrogen peroxide and peracetic acid treatments play a role in minimizing the hydrophilic nature of the fibers. The contrasting water absorption behaviors among the treated composites underscore the intricate relationship between surface modifications, chemical treatments, and the resulting material properties. While the NaOH treatment increased the water absorption due to the exposure of OH- groups, the hydrogen peroxide and peracetic acid treatments, despite minimal changes in FTIR spectra, influenced water absorption through surface roughness and potentially enhanced adhesion.

**Figure 5:**
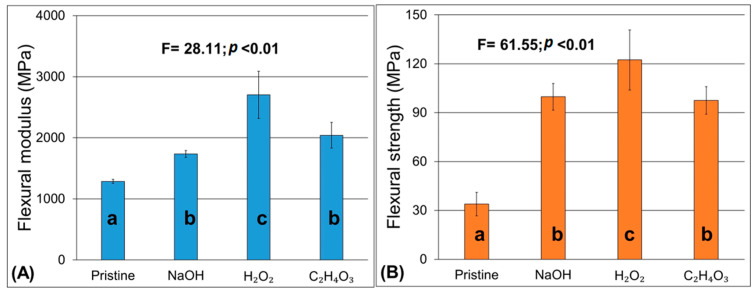
Flexural properties of the jute/polyester composites: (**A**) flexural modulus and (**B**) flexural strength.

Figure 5 shows the effect of the fiber treatments on the flexural strength of the samples. These samples were manufactured as we explained in Section 2.3. All treatments proved to be effective in increasing the flexural strength and modulus of the composites, with hydrogen peroxide being noticeably effective. This is in line with the water absorption results herein found, which could indicate better adhesion at the fiber/matrix interface and, therefore, increased stress transfer at the interface, leading to better flexural properties. For mercerized fibers (i.e., treated with alkali), even though there were some changes in the surface chemical composition, as evidenced by the FTIR results, the changes were not as pronounced as when the other treatments were used, although an improvement from pristine fibers was found.

Chemical treatments play a crucial role in enhancing natural fibers by removing unwanted elements like waxes, oils, and dirt from their surface. Additionally, these treatments increase surface roughness, facilitating mechanical adhesion. However, the effectiveness of chemical treatments varies among different fibers and even within the same species. Lavoratti et al. [23] investigated the mercerization process on buriti and ramie fibers using varying concentrations of NaOH solution. They observed that while mercerization improved the flexural strength of ramie fibers, excessively high concentrations could lead to fiber degradation and a decline in mechanical properties. Conversely, fibers with higher lignin content, such as buriti fibers, can withstand mercerization at higher concentrations, with only minimal impact on the resulting composite’s mechanical performance [23].

**Figure 6:**
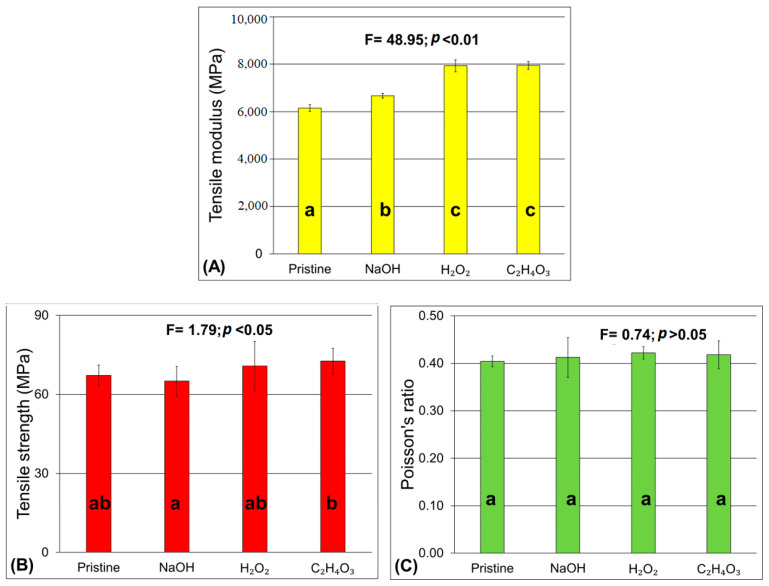
Tensile properties of the jute/polyester composites: (**A**) tensile modulus; (**B**) tensile strength and (**C**) Poisson’s ratio.

The results for the tensile strength, modulus, and Poisson ratio of the composites are presented in Figure 6. The tensile modulus increased when chemically treated jute fibers were used. When H_2_O_2_ and peracetic acid treatments were used, an increase in the rigidity of the composites was observed, and the tensile strength was comparable to NaOH-treated composites. These results could indicate that the fiber/matrix adhesion was effective until the formation of permanent mechanical damage in the composites. Sever et al. [32] studied the properties of jute fabric/polyester composites treated with sodium hydroxide and observed a marginal increase in the tensile strength for the treated fibers in comparison with untreated ones. The authors reported an increase in this property only when other treatments were used in conjunction with the alkali treatment and attributed this behavior to the added hydrophobicity promoted by additional treatments, increasing the compatibility of the fibers with the matrix.

**Figure 7:**
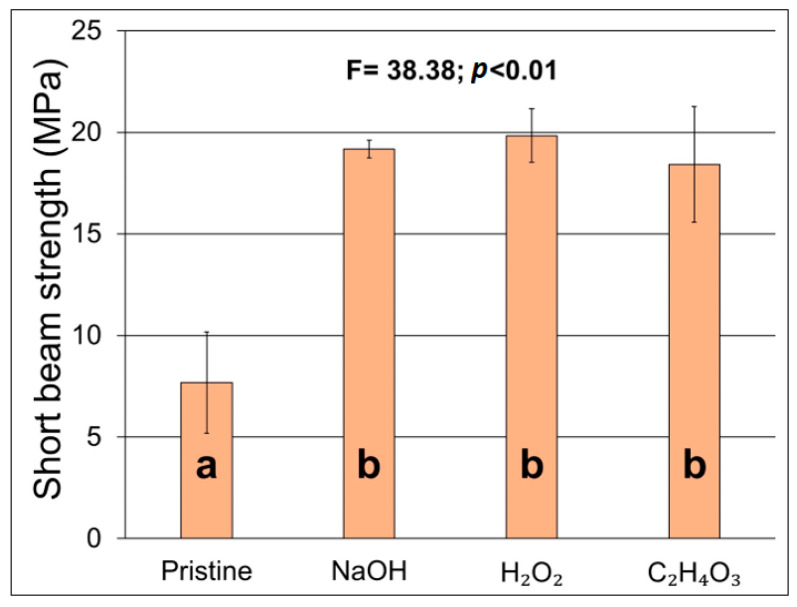
Short beam strength of the jute/polyester composites.

The short beam strength is presented in Figure 7. It is possible to observe that all treatments were equally effective in increasing the shear strength of the composites in comparison to the neat jute fiber composites. Moreover, there was no significant statistical difference among the treated fiber composites, which could indicate that even a small gain in adhesion was sufficient to enhance the short-beam strength of the composites. Da Silva et al. [33] also reported an increase in this property when treating sisal and curaua fibers. The authors also noted that the optimization of the treatment by varying the time, temperature, and concentration of the solutions could improve the fibers’ surface and, in turn, enhance the properties of the resulting composites.

Overall, the chemical treatments resulted in varied mechanical responses, albeit significant in some cases, e.g., the flexural modulus and the interlaminar shear strength in comparison to the untreated jute fiber composite. In addition to that, no chemical treatment promoted a universal increase in all properties evaluated in this study. It is important to note that, while chemical treatments may enhance the adhesion at the fiber/matrix interface by means of removing impurities or increasing the fibers’ surface roughness, as some authors reported [12], the effectiveness of such treatments depends on the concentration of the solution, time, and whether one or more treatments were used in combination with one another. Moreover, as natural fibers have varied compositions depending on the harvest or the species, chemical treatments do not universally respond the same across the studies available in the literature.

**Figure 8:**
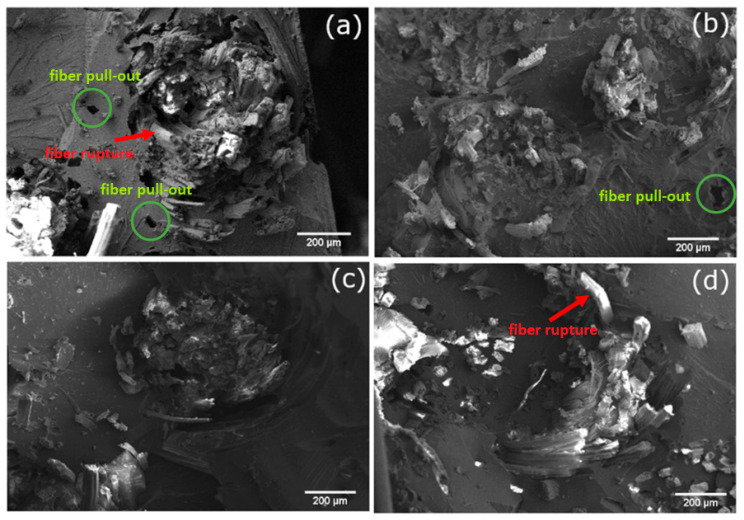
SEM micrographs of the composites with: (**a**) neat jute fiber; (**b**) NaOH-treated jute fiber; (**c**) hydrogen peroxide-treated jute fiber and (**d**) peracetic acid-treated jute fiber (200×).

The SEM micrographs of the composites are presented in Figure 8. In all micrographs, it is possible to observe some agglomeration of the fibers, which could have hindered some mechanical properties in this study. Figure 8a,b, representing the neat jute and NaOH-treated jute fiber composites, show some points of fiber pull-out, which are related to poor adhesion at the interface [34]. The fiber pull-out is more evident for untreated fibers, and it corroborates the mechanical strength results herein found. Untreated jute fiber composites also show some points of low adhesion, probably due to the lack of surface roughness seen in Figure 2a. Figure 8c,d, which show the composites with hydrogen peroxide and peracetic acid-treated jute, show fewer points of fiber pull-out and some places where fiber rupture occurred, which is a sign of good adhesion [35] and, thus, enhanced mechanical performance. Some evidence of fiber pull-out is highlighted using green circles in Figure 8a,b. Additionally, some visible occurrences of fiber ruptures are denoted by red arrows in the figures, which is a indicative of favorable adhesion.

The introduction of surface roughness, as evidenced in Figure 2c,d, may contribute to the enhanced adhesion and mechanical performance of these treated jute fibers in the composites. The reduction in fiber pull-out points suggests that the chemical treatments have improved the interfacial bonding between the jute fibers and the polyester matrix [35].

Overall, the SEM micrographs provide visual evidence of the impact of chemical treatments on the morphology and adhesion characteristics of the jute/polyester composites. While untreated and NaOH-treated fibers exhibit some points of poor adhesion and fiber pull-out, composites with hydrogen peroxide and peracetic acid-treated fibers demonstrate improved adhesion, as indicated by fewer pull-out points and instances of fiber rupture. These findings align with the mechanical performance results, emphasizing the critical role of chemical treatments in optimizing the interface and, consequently, enhancing the mechanical properties of jute/polyester composites.

## 4. Conclusions

Jute fabric composites with both pristine and chemically treated fibers were successfully obtained by RTM. The treatments induced changes in the fibers’ morphology, and the alkali treatment was effective in removing some lignocellulosic contents. Even though adhesion improved, as seen in the composites’ micrographs, water absorption increased for NaOH-treated composites, showing that despite being an effective treatment, higher water absorption could be a problem for the application of NaOH-treated composites.

The chemical treatments resulted in increased flexural and short-beam strength performance; the tensile properties did not change remarkably. Some fiber pull-out was observed for both neat and NaOH-treated jute composites, but hydrogen peroxide- and peracetic acid-treated jute composites showed good adhesion, better mechanical performance, and fewer points of fiber pull-out, along with lower water absorption.

## Data Availability

The study did not report any data.
